# Choreographies of sexual safety and liminality: Forensic mental health and the limits of recovery

**DOI:** 10.1016/j.ssmmh.2022.100090

**Published:** 2022-12

**Authors:** Paula Reavey, Steven D. Brown, James P. Ravenhill, Zoë Boden-Stuart, Donna Ciarlo

**Affiliations:** a London South Bank University, 103 Borough Road, SE1 OAA, London, United Kingdom; b (Nottingham Trent University), United Kingdom; c University of Brighton, United Kingdom

**Keywords:** Forensic mental health, Sexuality, Vitality, Recovery, Liminality

## Abstract

Medium secure forensic psychiatric units are unique environments within the broader “post asylum” landscape of mental health services. Length of stay is much greater and restrictions on behavior, including sexual behavior, are legally and institutionally legitimated, due to concerns regarding risk. As a result, sexuality is rarely explored experientially with service users and no official policies on sexual conduct and sexual safety have yet been developed.

The aim of this study was to explore with service users how they experience and more specifically “feel” their sexuality during their time in secure care and in the community. A further aim was to understand how sexuality connected with their thoughts and feelings on recovery and relationships and their perceived impact on mental health. We report on the findings from 29 service users participating in a qualitative-visual study, using drawing as a visual technique to provide an opportunity for expression of feeling.

In this paper, the analytical focus is on how institutional practices can induce a “liminal hot-spot”, wherein an impasse between past crisis and future recovery is reached, taking theoretical direction from Stenner's work on liminality and Fuch's work on vitality. Specifically, we examine how service users experience liminality and the practices that emerge from such a state of living that can serve to objectify and suspend feelings of vitality. Finally, we discuss the implications of these findings for a recovery model and the development of policies on sexuality, sexual safety and relationality, within secure forensic mental health settings.

Issues of sexuality in secure mental healthcare have been overlooked historically in both clinical practice and the academic literature (Brown et al., 2014; Hunter & Ahmed, 2016; McCann, 2010; Ruane & Hayter, 2008). However, as many as 30% of people in mental healthcare report participating in sexual activity, often in contravention of policies banning such contact (Warner et al., 2004). Furthermore, research findings suggest that fulfilling intimate and sexual relationships may be associated with positive adjustment to life in mental healthcare services, in the community after discharge, and with other positive mental health outcomes (Gilburt et al., 2008; Kawachi & Berkman, 2001). Sexuality is essential to the human condition, a human right (Hicks, 2016), a fundamental aspect of “the totality of being a person”, and an important component of recovery (McCann, 2000, p. 134). Moreover, sexuality can offer a sense of vitality, as “a manifestation of life, of being alive” (Stern, 2010, p. 3), which can interleave with feelings of hope, and in turn, recovery (Deegan, 1999). How can inpatient sexuality be managed in secure mental healthcare facilities and what kinds of evidence-based policies might be designed to ensure that inpatient sexuality is addressed appropriately? The purpose of this study is to provide insight into lived experiences of inpatient sexuality in a secure mental healthcare facility (hospital) located in England^1^. A key concern is how patients manage issues of sexuality on an experiential level and how such experiences intersect and are caught between their treatment for mental ill-health, hospital practices and policy. The study adds to the call for institutional and national policies on incorporating considerations of sexuality as part of the care plan of people who live in secure mental healthcare.

The Five-Year Forward View for Mental Health report produced by the independent Mental Health Taskforce for NHS England (2016) presents a clear set of challenges for promoting recovery in secure mental health services. It points to a pattern of stable admissions to inpatient care but with increasing severity of needs and rising numbers of persons with “complex needs” being detained. The report calls for care to be “safe, effective and personal and delivered in the least restrictive setting” (p.9) but nevertheless concedes that a lack of consistency in the provision of secure mental health services, long stays, a lack of step down or transitional services, and a lack of recovery-focused care has hindered this. Central to this report is a tension between the known benefits of increasing patient “personalization” and the capacity afforded by services to establish the necessary “equal and collaborative relationship” (p.43) that will facilitate this. Part of this personalization agenda highlights the importance of healthy and stable relationships, the re-establishment of intimacy and the recognition that positive relationality can assist with long term recovery. However, secure mental healthcare facilities typically prohibit sexual or intimate relationships among inpatients (Bartlett et al., 2010; Brown et al., 2014; Deegan, 1999; Ravenhill et al., 2020), justified within discourses that frame inpatients’ desire for intimacy as a distraction from treatment, and an antecedent to unintended harm (Hunter & Ahmed, 2016; Ruane & Hayter, 2008).

Despite the recognition that a significant minority of patients do form relationships and engage in sexual activity in secure settings, official advice for clinicians on this matter is not forthcoming. The Royal College of Psychiatrists' (2017) report on sexual boundaries draws attention to inpatients' rights under the European Convention of Human Rights (ECHR) to pursue romantic relationships, while noting that, according to ECHR, “[clinicians'] interference” may be warranted, for the “protection of [inpatients’] health” (p. 11). Although decision making falls under the remit of responsible clinicians, the report does not offer any guidelines on how such decisions relating to the facilitation or prohibition of patient sexuality might be reached. The unintended consequence is for patients to be “caught between” not being directly prohibited from engaging in sexual activity, but with no real sense of how this sexual expression might be achieved, within the confines of clinical decision making and hospital policy.

This experiential mode of being caught “somewhere in between” is referred to here, following Stenner et al. (2017), as a “liminal hotspot”. A liminal hotspot is an occasion during which people feel they are caught suspended in the circumstances of a transition that has become “stalled” (Motzkau & Clinch, 2017). The patient is suspended from “normal life” due to containment in a hospital setting, with attendant practices and rules that follow. Life, as it was known, thus becomes suspended, with many feelings, pleasures and memories put on hold. Brown and Reavey (2015) have referred to this “suspension” as a mode of “presenteeism”, whereby desires, feelings and memories are deferred and displaced in favor of a persistent concern with what is happening in the present – with foremost attention paid to present behavior and the level of risk observed, especially where sex is concerned. The institutional concern with the present and “what can be seen”, and more importantly, what harms can be averted in the here and now, can filter through to service users’ experiences upon discharge, where expressions of sexuality are treated with suspicion, caution and fear (Brown et al., 2014; Ravenhill et al., 2020): One may no longer reside within the confines of the walls of the unit, but the opportunity to resume a “normal” sexual life can prove difficult. It is this liminal state which serves as a potential impediment to the necessary movement required to facilitate relationships and sexuality in institutional and community living. In the next section, we show how the concept of liminality can cast theoretical light on the issue of sexuality for secure inpatients, by further connecting it to the concept of vitality.

## 1 Life, liminal hotspots, sexuality, and mental health

Medium and low secure mental health facilities are designed as therapeutic spaces that serve as a means of stabilising acute distress and facilitating long-term recovery from mental ill-health. Such facilities are charged with providing round the clock psychological and physical safety, and effective, evidence-based treatments under the supervision of trained mental health professionals. Rather than mere containment, the purpose of these spaces is to restore and rehabilitate, in preparation for transition to community life, or return to prison. Despite this ambition, a fair proportion of inpatients report substantial difficulties with secure environments, ranging from staff indifference and a desire for prison over hospital life, through to confusion and hostility regarding the purpose of long-term detainment (Reavey et al., 2017; 2019). The following quote from a participant in a study by Brown et al. (2014, p. 250) captures some of the ambivalence mental health inpatients can feel:

I would say this place has amputated my sexuality. Definitely, it's not my home, it's not a free environment and it's so anti-life. I just don't even think about sexuality in here and I grieve over that quite a lot. And I try and cope with this place on its own terms, you know and whatever it has to offer me I will engage with. So I try to make it a reality, its own reality but I still can't feel human enough to be a sexual being in this environment (Anne)

The participant expresses how the lack of freedoms and restrictions have impacted upon her relationship to her sexuality and her broader sense of her “life”. She talks of her sexuality as being “amputated”, cut off from her current experience because it cannot manifest in the current environment. She sums up her feelings about the space where she is currently detained as “anti-life”. This idea can be elaborated in relation to the concept of “vitality” developed by Daniel Stern (2010) and Thomas Fuchs (2013), who emphasises that good mental health is rooted in a “prereflective, undirected bodily self-awareness that constitutes the unnoticed background of all intentional feeling, perceiving, or acting” (p. 2). “Feeling alive” underpins a sense of engagement with others and with the immediate environment. Vitality is not a continuous tone, but rather fluctuates and wavers. As Stern (2010) describes, vitality follows patterns of escalation and de-escalation, crescendos and lows, expansions, and contractions. Vitality is interrupted, constrained, and then released. Rather than seeing these constraints on vitality as external to and imposed on “life”, Frederic Worms (2015) argues that constraint and resistance is internal to the nature of living itself. For Worms (2015), “critical vitalism” is the recognition that life – the vital – needs to undergo a continuous closure in the form of turning around on itself, and reorganization – the critical – to open up differently, thrive and develop. On this basis we can characterise what Anne describes above as a space that supports only the closing down of life, and not its opening up.

If sexuality is placed within the broader framework of vitality, as reflected in the participant quote from Brown et al. (2014) above, then the issue is not whether inpatients should have the freedom to express openly an unrestrained sexuality, but rather how to prevent it being closed down altogether. Closure has the effect of interrupting and suspending the relationship inpatients have with their own sexuality, with implications for their broader recovery journey. Sexuality becomes an object of uncertainty, an aspect of the self – positioned in discourses of risk and danger – that should be forgotten, concealed, and perhaps entirely deadened by pharmaceutical treatments (de Jager et al., 2017). The participant is effectively caught between two worlds and two periods in time: They are neither at home nor able to imagine the feelings which they might experience when they are able to exit this “anti-life” environment.

The concept of liminality captures this sense of being caught “betwixt and between” a past that has ended and a future which is yet to begin. Originally developed in the anthropological work of van Gennep (1909), the concept was significantly reformulated by Victor Turner (1964) and has been developed further in contemporary psychosocial approaches (Stenner, 2017; Thomassen, 2016). Here, we use the concept of liminality to underline how secure care suspends the movement in mental health from “crisis” to “recovering” by enforcing an interregnum between being sexual to non-sexual upon hospital admission, and in some cases, back again upon discharge. This creates both an *impasse*, where the interruption of the everyday, taken for granted situation becomes permanent, and a *paradox*, where patients are meant to strive for the vitality of recovery whilst being denied the possibility of expressing the intimate and relational feelings that are intrinsic to a sense of recovering.

Stenner et al. (2017, p. 142) refer to situations of ongoing psychosocial impasse as liminal hotspots:

[A] liminal hotspot does not refer to an observable object: it is a happening, rather than a thing; an event, rather than an entity. It does not passively wait for us to describe it, rather it *occurs* as an emergent feature of the play of circumstances: circumstances in which the usual normative orders are for whatever reason suspended or disrupted

The dominant experience of a liminal hotspot is the sense of perpetual suspension. The open-ended nature of secure mental health care, where there are no specified limits as the length of inpatient detention, already provides the conditions for this experience. But the central problem here is the paradoxical injunction levelled at the patient to achieve wellness through rejecting aspects of what they were, whilst being prevented from experiencing those feelings which may help them become someone “in recovery”. The difficulty with this kind of liminal hotspot is that the central paradox is often not acknowledged or integrated into the treatment regime or long-term care plan of the patient, which can lead to confusion, fear, and stalemate. Paradoxes can be resolved spatially in fairly simple processes (patients can be separated, isolated, and treated on site); in complex processes, such as the transition between hospital and community; however, such paradoxes are not easily concluded, especially if the liminal hotspot of the hospital transfixes the individual, because they have not been given the “permission” to open up possibilities for action. Thus, a liminal hotspot is perpetuated when a situation cannot be *de-paradoxified*, or one cannot escape from the confusion between action and prohibition. In the case of sexuality in secure mental health care, the paradox is difficult (but not impossible) to disrupt. The societal directives of security/detention/confinement/safety versus care/cure/growth are thus central to how such hot spots emerge and potentially remain firm within the units, since the paradox lies at the point where evidence-centred and patient-centred logics of care (and risk aversion) collide and create mutual interference. In these terms, patient sexuality can all too easily result in a practice stalemate.

## 2 From risk management practice to choreographies of sexual safety

Many European nations have only informal or local policies and practices in place which cover the expression of sexuality and patient relationships in secure care. In a study of secure care in 14 European nations by Tiwana et al. (2016), many of the expert respondents surveyed stated that very few problems associated with the existence of policies had emerged over the decades in which they had been in place. There are also outlier examples, such as Italy, where the decision to shift entirely away from secure mental healthcare has removed the conditions under which liminal hotspots occur (Barbui & Saraceno, 2015; Vorstenbosch & Castelletti, 2020). The situation in the UK is complexified by the overwhelming focus on risk management within secure settings (Jacob & Holmes, 2011), and particularly on the embedding of this within the physical environment or “technical safety” (Curtis et al., 2013). For instance, recent efforts to address inpatient sexuality have focused primarily on ensuring “sexual safety” from predatory behavior and non-consensual sexual acts between patients (Care Quality Commission, 2018). Although such considerations are clearly important and part of the duty of care of institutions, they may serve to mask a broader sense of sexual safety, as the relational practices members of a community adopt to keep one another safe.

Mental health care institutions within the UK are themselves then caught in a paradox. Faced with a national policy of proscribing sexual activity whilst confronted with the inevitability of intimate relationships between inpatients, there is a tendency to either delegate responsibility to one part of the organization (such as social workers) or to devise local ward-based practices (Poole, 2020). This predictably leads to accountability being shifted around the organization rather than wholly owned, and to less senior and more precariously employed staff (such as bank nursing and health care assistant staff) assuming a large part of the risks involved in managing patient sexuality, with concomitant inconsistencies in practice (Ravenhill et al., 2020). The absence of clear “top-down” policy also tends to make ward staff more risk-averse in the way they approach patient sexuality, and more likely to see sexual activity as instances of “organizational misbehavior” or deliberate rule-breaking, rather than as attempts by patients to resolve the liminal hotspot in which they find themselves (Ravenhill et al., 2020). But linking the paradox of the institution to that of patients can be done productively. The route to deparadoxifying institutional problematics lies in i) better understanding and openness to the emergent deparadoxifying strategies of patients and ii) rethinking the ways in which these strategies are objectified in broader organizational processes.

Liminality presents an opportunity for growth and transition. The constraints which prevent persons from immediately transitioning between two phases of existence provide the necessary provocation for creative transformation (Rosaldo et al., 2018; see also; McGrath et al., 2021). Patients can and do find ways to express their sexuality that pass “under the radar” of staff and institutional concern (Ravenhill et al., 2020). These emerging attempts to deparadoxify the liminal hotspot patients find themselves caught within can be considered as openings which may support growth and recovery. Of course, other emergent practices may be damaging and unhelpful, but without an openness on the part of the institution to address these attempts, there is no possibility of helping patients to distinguish either way. Key to this is the common tendency amongst ward staff and clinicians to treat expressions of sexuality as a “sign” of underlying mental health instability. For example, as researchers we have in the past been cautioned by clinicians to be aware that 'female' inpatient participants may “act out” sexually during research interviews. Further, staff may “veil” patients’ sexuality in response to their own fears around competence, confidence, and professional vulnerability (Higgins et al., 2008; Quinn et al., 2011). We argue that the meaning of sexuality needs to be attended to as it is in process of being created, rather than something that is already known and arrived at and then sanctions/treatments applied to. The point then is to understand the choreography of sexuality at play, while liminal hotspots are in the process of being navigated by patients and staff and negotiated within those institutional relationships and sets of practices.

There is no openness without accountability. When emerging practices become visible, they also inevitably become objectified as matters of institutional concern. Charis Cussins (1996) contrasts two senses of objectification in clinical settings. The dominant sense is that patients are necessarily “disciplined subjects par excellence” (p.578) in that their agency is subordinated to classificatory practices on the setting in question. In secure mental healthcare, this would involve a reduction of life experience to the diagnoses formally assigned to an inpatient. But it is also possible, Cussins argues, for objectification to both enable and support agency when it is carefully organized in relation to a longer-term project of personhood. In fertility clinics this is possible when the various objectifying physical procedures that women are subjected to are metonymically connected to the desire to become pregnant. Cussins refers to this as “ontological choreography”, the co-ordination of procedures, techniques and actors which is in the service of developing the transformation of the person towards some valued goal:

The choreography is the coordinated action of many ontologically heterogeneous actors in the service of a long-range self. The treatment is a series of interventions that turn “where is it broken?” into a well-formed way of asking “why aren't you pregnant?” (Cussins, 1996, p. 600, p. 600)

The parallel move which could be made in secure mental health care is to turn the question “what is your sexuality telling us about what is wrong with you?” to “based on your life experiences, how might sexual expression offer opportunities for growth and recovery now and in the future?". The choreography of sexual safety envisaged in posing the latter question would involve a necessary objectification, but it could take the form of careful elicitation of sexual needs and desires, openness to engage with emerging practices, and multiple organizational procedures for supporting sexuality through a recovery-oriented focus. In the subsequent analysis section, we will begin to address these issues by exploring i) how mental health service users relate to their sexuality and the way it is currently institutionally managed; ii) the kinds of emergent practices for deparadoxification that service users develop; and iii) what forms of objectification are currently enacted and what effects these have in relation to longer-term recovery. In the discussion section we will build upon this to sketch out an agenda for policy and practice around choreographing sexual safety.

## 3 Method

The qualitative material analysed here was collected as part of a broader Wellcome Trust-funded project conducted within a large, purpose built, medium-secure forensic mental health unit and in community safe houses, in the UK. All the community service users had at one point been inpatients in a medium secure unit and had made the transition to community life, via low secure and rehabilitation units.

The hospital inpatient unit was located within a large well-established hospital site, which includes a wide range of other psychiatric and elderly care units, including other locked wards and low-secure pre-discharge wards. The overall aim of the study was to examine how service users experienced their sexuality whilst in hospital and in the community. The project was concerned with capturing the feelings and lived experience of service users, so attention to rich description was central to how the interviews were conducted. The research was primarily based around interviews with service users, along with observations recorded during the periods of fieldwork. Observations were recorded in researcher diaries, and then used to supplement interview material where relevant. A heavily descriptive participant profile was created for each service user, charting details of their past experiences and life histories, to provide further context to the overall themes reported in the analysis. Observations relating to staff and patient movement, behavior and the overall atmosphere of the ward were also registered, either during or post visit. In addition to using the term service user, we use the term “patient” to describe those participants who were detained in secure care within a forensic pathway. Whilst this term is technically accurate, we are aware of the problems with this term and in other contexts would refer to “individuals who use services” or “individuals who live with distress” (see Cromby et al., 2013).

The specific research reported here is drawn from 29 interviews with inpatients (21), and 8 community service users. Each interview was conducted by four of the five authors, lasting between 45 and 90 ​minminutes, supplemented by observations of ward practices (see above). Before access to patients or staff was permitted, permission was granted by the local NHS and London South Bank University research ethics committees.

The interviews used a drawing methodology (Boden & Eatough, 2014; Reavey & Brown, 2021) to elicit more specific and rich responses relating to sexuality, sexual feeling, and mental health. This visual-qualitative approach has been used in the context of examining experiences of distress, including first episode psychosis, to elicit metaphorical, symbolic, and difficult to reach feelings (Boden & Larkin, 2020). This approach affords a more direct engagement with the phenomenological detail of feelings, which can be hard to articulate in interviews (Reavey, 2021). Participants were asked to draw anything, either literal or abstract, that captured their sexuality. They were guided by reassurances that the drawings did not have to be of anything concrete, could be metaphorical or symbolic, and did not have to be technically proficient. Participants were offered a range of materials to choose from, including colored pens, paints, and crayons.

The interviews followed a semi-structured format, to the extent that a schedule developed by all researchers was used to guide the conversation. However, the interview was guided primarily by the participant's engagement with the visual material, such that the order of questioning was led by the participant's discussions via the drawings they produced prior to interview. Overall, participants engaged with the visual material and interview questions well, with varying levels of engagement with material of a more personal nature. A very small minority refused to draw, but the interview went ahead as planned, using the same interview schedule. Each interviewer agreed in advance that the interview would be participant led, with questions being addressed at the participant's pace.

Participants were asked to discuss the drawings, in terms of their thoughts and feelings about their illustrations of sexuality and sexual feeling, as well as their experience of the hospital and/or the community more generally. The use of visual material alongside verbal data is advocated within a growing body of work in psychology and psychosocial research (e.g., Reavey & Brown, 2021; Reavey & Johnson, 2017; Rose, 2001). Visual materials are typically thought to provide more effective prompts for participants to discuss the settings and context of their experiences, since they contain clear spatial cues (see Bolton et al., 2001; Knowles, 2000a; 2000b; Reavey, 2011, Reavey, 2020). In this research, the drawing technique was intended to support participants articulating aspects of their experience that might be difficult to put into words, such as feelings associated with sex and embodied experience (see Boden & Eatough, 2014; Brown et al., 2011; Gillies et al., 2004; 2005). The method was used to empower participants with regard the structure of the discussion and to offset some of the well-known effects of medication on the interactional abilities of psychiatric patients by providing a clear point of visual reference.

The interviews were digitally recorded and transcribed verbatim. (One participant did not consent to their interview being audio-recorded. In this case the interviewer recorded hand-written notes.) The participants' names were replaced by pseudonyms, chosen by the researcher. The drawings were given meaning by the participant only, in the context of the interview, rather than treated as data to be analysed independently (Reavey & Prosser, 2012). The authors’ analytical reading of the audio material was guided by the overall research question: how did participants experience sex, sexuality, relationships, and sexual feelings during their time in hospital and in the community? The main aim of the interviews was to provide the space for participants to express feelings relating to sexuality, including feelings about their bodies, other people, as well as how they felt about sexuality in relation to their recovery. The visual-verbal mode of expression intended to encourage articulation that stretched beyond “talking about/around” sex and sexuality, and more effectively invited rich description of specific feelings and thoughts, located in the body and in space (see Reavey and Brown (2021), for further discussion).

After notating and coding the material with these questions in mind, the data were re-organized into themes and subsequently considered in the light of literature that could assist in contextualizing the analysis. A thematic decomposition (Stenner, 1993) approach was used to analyze the data, which sought to identify processes through which agency was understood and experienced, located in particular themes around space. This thematic decomposition was achieved by following several stages of analysis that are commonly found in many forms of qualitative analysis (Willig, 2008). This involved familiarization with the data via repeated readings of the transcripts, generating initial codes by paying close attention to meanings embedded in every line of talk, followed by matching the initial codes together to form candidate themes and sub-themes, with the research questions as organizational guides. Each of the authors was involved in discussions around whether the generated theme titles and definitions adequately captured the essence of the data.

The analysis that resulted was “theoretical” insofar as a concern with the constitution of vitality and liminality was present from the initial reading and notation of the data. Nevertheless, the interpretation produced was also “inductive”, in the sense that the final account produced was based on a close reading of the experiential material (data). The interpretative process further involved exploring the implicit meaning of the material, rather than a more descriptive reading. The validity of the findings was addressed using conventional qualitative procedures, including group analysis by key researchers and peer review, to ensure the analysis was sufficiently grounded in the data (Creswell & Miller, 2000).

## 4 Analysis

Our analysis identified several institutional practices within the forensic mental health unit that shaped how sexuality was constituted for and experienced by patients. The analysis covers participants in hospital and in the community, as we wish to examine more closely the ongoing relation between the institution and the community, as we have noted previously that the (psychological, physical and social) influence of the hospital does not necessarily cease upon discharge (Brown et al., 2014). We focus here in the analysis on experiences that open possibilities for sexual expression, as well as those which seem to point to a closing off or deferral, displacement or closing down of sexual expressions and encounters. The central issue here concerns the interplay between how sexuality is caught between a set of competing practices at an institutional (policy) and clinical (treatment) level, which is then experienced by patients, as confusing and contracting of agency, and potentially recovery (see also Reavey et al., 2019.)

## 5 Waiting in suspension and the diminution of life

Many of the patients described thinking about sex either in terms of the present, or thinking about what they were able to do in the past. Most of the participants had never spoken with staff about their needs, feeling that such a conversation was either not possible, or would lead to further scrutiny and perhaps restriction, constituting a “reductive objectification” in Cussins’s (1996) sense. The drawings produced in the interviews provided a means of expressing feelings of sexuality, literally or metaphorically, using various colors to depict their relationship to their sexuality. One recurring theme was how sexuality had to be suspended, until such a time as the patient was able to leave hospital and live elsewhere. This metaphorical connection between the suspension of sexuality and the diminution of life is captured in the extract below:

[My sexuality] is all about different shades of pink.

I: Is it still pink while you're living here?

At the moment it's probably like a blue, because you can't really do anything.

I: Why blue?

It's a sad, dull color isn't it?

In hospital, Megan's sexuality turned from different shades of pink – depicting variation and vibrancy – to cold blue, suggesting a suspension of vibrancy and heat: Life lost its color. Rob used the metaphor of a hibernating solitary animal, whose habitat is frozen and barren to describe his sexuality:

I: If your sexuality, the sexual part of you was an animal, right now what would the animal be?

A polar bear. It's winter, I'd be hibernating.

**Fig. 1:**
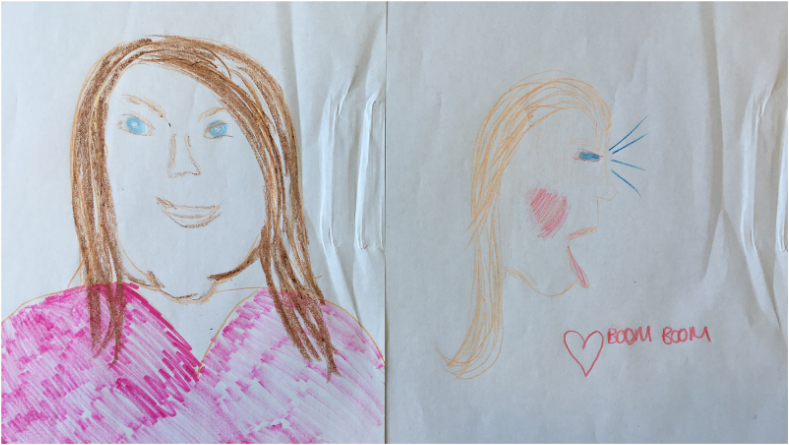
Megan's drawing].

Hibernation implies dormant life, waiting for an environment or period that can re-ignite (sexual) appetite. Sexuality remains a part of the person, but it is entirely closed up, unavailable as part of the experience of secure care, and hence not a source of growth and recovery. Josie referred to her sexuality as a “rosebud, not open,” again conjuring an image of sexuality as present, ready to burst with life, its potential unrealised due to hospital living conditions. This notion that patients can be caught in a paradox of unrealised potential sexual energy is clear from a number of the interviews. For some, this resulted in a deferral of sexuality, in favor of “getting better” and leaving the institution because this is what they had been encouraged to do by their clinicians and felt it would cause less distress (see also Brown et al., 2014) (Fig. 1). In part, this was encouraged by institutional risk discourses, which emphasised the need to “behave” and “stay focussed” on reducing vulnerability and risk by abstaining from relationships, especially with other patients:

I was trying to date women in hospital but the reason I didn't go forward with it, because I didn't want to look … what's the word … I didn't want to look at a person and think, “Well, you're the same as me. You're unwell. Maybe it wouldn't work out. Maybe because you're unwell like me you're confused and that.”

Joseph characterised his unwillingness to engage in personal relationships with other inpatients as based on the mutual recognition of being mentally “unwell”. Sexuality was seen as perpetuating poor mental health rather than a step towards recovery. As such, it had no place within the hospital. But this moratorium on sexuality means that if it cannot be addressed within a therapeutic setting, it simply becomes something that patients such as Joseph will need to work through by himself at some unspecified later date, rather than being brought into his recovery process at a time when it might be most relevant. This “paradox of potential” can further result in a sense of being caught suspended, leading to frustration and stalling:

If I'm frustrated already, you're not going to be able to move on are you? If they say, “You can't do this, you can't do that,” you get more frustrated. And by the time you leave you're so frustrated you don't know what to do with yourself.

According to Hayley, the refusal on the part of the institution to address sexuality acts as a direct obstacle to recovery and is dis-enabling of her ability to imagine their future life. Other participants dealt with the frustration of their suspended sexuality by dis-associating their thoughts and difficult feelings, to secure their discharge from hospital and to manage feelings:

Because even if I think about it, even if, say, there's someone I like, there's nothing I can do about it that isn't going to be detrimental to my progress as a patient working my way through the system.

Once again paradoxically, such a suspension of sexuality induced feelings of hopelessness (an antidote to recovery) and negative feelings and low mood, for several participants. This sense of holding feelings, but being denied access to them, for some was a paradox that was difficult to bear, at the level of feeling:

I don't like to think about it because of the way it can make me feel bad, you know. I try not to think about it too much because I just get depressed, I get down or I get upset or angry, because I know that, like, I might feel lust or maybe even more than lust, but I can't act on it. And having those feelings and knowing that you can't act on it can be quite hard.

Some referred to this containment of sexual feeling as corrosive and for some even dangerous, referring to their sexuality as a “ticking time-bomb” or something that would “kill them”, like a “bee releasing its sting”. The life and death discourses, deployed in several participants’ accounts are worthy of note, as once again we see how patients are not given the tools to deparadoxify, because their route to opening up their sexuality or having conversations about it are closed down, at an institutional level. The capacity to realise their potential, as sexual beings, is recognised as potentially harmful by both patients and clinicians, without opening the potential for more productive conversations about sexuality and indeed sexual safety. Being stuck (in a liminal hotspot) is thus the result of not providing the means of working with patients collaboratively to establish and enhance the *life-giving potential* of sexuality.

## 6 Objectification and the purification of sexuality

To manage the paradoxes of sexuality whilst in hospital and in the community, participants turned, often in fantasy rather than reality, to take ownership of objectifications which they felt originated both specifically in the secure setting and in more general culturally embedded discourses around sexuality. For example, one strategy used was to not only accept the current prohibition on sexual activity, but also to see it as a temporal marker where prior experiences of sexuality belonged to a past that was definitively over:

I was thinking about coming out of hospital, getting a good man… But for now, I feel like a clean person.

I: A clean person?

Yeah. It makes me feel good about myself, that I'm not having sex.

Emilie made use of a purity/dirty binary to deparadoxify her current feelings: Sex belonged to a former, “dirty” self, and purging herself of sexual thoughts and feelings was a way to feel like a better, “clean person”. The dirt/clean dichotomy operating in this participant's discourse once again reinforces the notion that if sex is eliminated then order will follow, as purity of mind and body facilitate wellness and self-esteem. This kind of deparadoxification appeared to be effective for Emilie in that it reduces the liminal tensions around sexuality, but we might have cause to question whether it is an effective long-term strategy in service of her recovery, given that any potential “dirtiness” she is exposed to later might be experienced as a threat to her mental health. Brown et al. (2014) refer to such relationships to self that are learned in secure settings as “psychologically modified experiences”, and similarly question how adaptive they may be when services users return to community-based mental health care.

The participants’ ownership of sexuality was often circumvented in favor of staff intervention in hospital, and even in the community, if the participant was in low secure accommodation. Matthew reflected on having to deal with the effects of a “forced separation” during his time in services, which led to humiliation and confusion over his status as an adult:

When they thought me and this lad were getting too close, it would be brought up in ward round. How humiliating is that? You'd have in a room like this, with four or five or six professionals, and there were asking you about stuff, you know, “What is going on between you and him?” and “Don't you think that, you know, it might be a sign that, you know, you're not so well, or perhaps it's not such a good idea,” or they actually said to us at one point, “We'd rather you didn't go out on leave together."

The ward round conversation here performs a reductive objectification where a growing intimacy is not handled as a potential source of meaning, but rather a sign of underlying mental health issues whose meaning is already known in advance. As previously mentioned, the focus on risk and abstention (not going out on leave together) denies the opportunity for open discussion around how to work with some of the positive feelings that might arise from the relationship, which could then be built upon as a way of choreographing safety between these individuals. A dialogical and collaborative approach, whereby the participant might openly discuss their apprehensions, joys, excitement and misgiving surrounding a relationship are closed down in favor of avoiding any such (potential) risk. The need for connection and tactility, both emotionally and physically, was described as an important facilitator of recovery for some patients, especially since some are in services for many years, with scant access to relational activity and any means to discuss sexuality and relationships. For many, the only option is to try to act like a “normal” person, by not giving in to sexual needs or even discussing them openly:

Because you have to work with the rules of life and ride the waves and ride your urges. As they said in DBT [Dialectical Behavior Therapy], you have loads of urges, you have to surf the urge. So I'm going to do [draw] a little surfboard here because I don't surf the urge. The reason why I didn't do our last meeting was because I actually went out and got drunk the other day. Which is not a good thing. So I don't-, but that was because of everything, relationships everything, because I don't sit with things too good. So what a normal person sits with, I actually don't. So I'm going to do a little surfboard down here, so I'm surfing the urge [draws surfboard]. I*: But you said that when it comes to surfing the urge, it's difficult?* It's very, very difficult because I cheat, and I don't mean to cheat… it's just an unwritten rule, you just don't talk about certain things. You don't talk about any urges ∗laughs∗ you don't talk about sex, you don't talk about past experiences, you just don't talk about them. So … and you've got an itch that you can never itch.

Metaphors of taming, riding waves and “taming the beast” were commonly used by participants as they described how they managed their sexual potential. What Claire called “surfing the urge” was a strategy for resisting objectification. She had learned that giving in to urges was “not a good thing” because she was not a “normal person” and could not risk talking about past experiences.

Personal relationships between patients do occur in secure settings. However, there is sometimes inconsistency in the ways these are approached by staff:

I've had a relationship with a man in a hospital and that just ended in disaster because the care team had to come and tell me that he wasn't the sort of person I should be sort of going out with. So they had to tell me, which is fine and, you know, “Thanks for a heads up”, but that didn't seem to be viewed as dimly as me being with my girlfriend was… He'd had a really long history of domestic abuse that I didn't know about, and that was the only reason they sought to try and split us up, whereas with my girlfriend it was just like, “This is wrong, you shouldn't be doing it and we're going to actually physically separate you.”

Staff attempted to choreograph Stella's relationship with a man through focusing on the patients' respective histories and prospects, whereas her same-sex relationship with a woman was handled by defaulting to an objectification of any sexuality as a risk. Outright prejudice towards participants whose sexuality did not fit with heteronormative ideals was not uncommon for participants, so it is unsurprising that heterosexual orientation was equated with “being well” and avoiding risk.

Sticking with the “norm” and meeting societal expectations was discussed as a way of being respectable in the community and earning the right to sexual freedoms. “Bad choices” when it came to negotiating sexuality constituted anything that contravened respectability. The point of suspension, of being caught in between normality and (ab)normality, is the lack of opportunity to confront sexual feeling, or even talk about what it might mean or look like, or whether it is even something desirable. This lack of questioning of the rules of normality meant that many participants felt that they would never be able to successfully sexually relate because they would never be in the market for normality. Thus, for many, normality was a fantasy because it was perpetually unobtainable and had to remain within the confines of the imaginary. Some participants believed that this was in part due to the lack of opportunity to engage in meaningful relationships with staff, so they had no point of reference to work with:

Just that I feel, I feel like coming in and out of hospital, being a vulnerable patient has stopped me from being a normal - having a normal relationship, because you don't have a normal relationship with staff, and you don't have normal relationships with anyone. And things are changeable all the time. You never know where you stand.

Rather than re-writing the rules on sexuality, or considering the complexity of their histories and some of the difficulties that might arise for themselves and others, the participants were left suspended in a liminal hotspot that left little opportunity to deparadoxify, little in the way of exchange relating to what might be done to open up possibilities, and thus, submerged in an uncertainty surrounding how to act.

## 7 Emerging practices of sexuality

There are other barriers to sexuality within secure care beyond those deliberately put in place by the institution. A common side effect of pharmacological medication for mental health is weight gain and disruption in the physiological aspects of sexual feelings. This can leave service users with acute feelings of being unattractive and feeling anxious about the prospect of sexual activity:

It strips you, it strips you of your masculinity, like, you put on tons of weight cos of the food and the meds.

… I've got a thing called delayed ejaculation… I think it's just from the anti-psychotic.

This reduction of sexuality to a specific aspect of embodiment is arguably a consequence of the kind of reductive objectification involved in refusing to address sexuality. Service users then focus on what they feel they have lost or are unable to do, rather than the role that sexuality might play in their future growth. However, for some participants it did at least provide a point of focus and indication of how they might address their current feelings of inadequacy:

I'm trying to rebuild my social activities but the main obstacle at the moment is my weight, because I put on a lot of weight in hospital… So I'm going to the gym and getting a good body again, and then I can start to have more sexual relations and that sort of thing… I used to get a lot of compliments on my body because I had a six-pack and I was really slim, but now my body's not so good. I wouldn't want to date somebody the same weight as me, so I need to get into a better place before I go and find somebody who I want.

Owen, who was living in a low-secure setting, saw his priority as “getting a good body again” through weight loss as a precursor to resuming sexual relationships. For him, the paradox of potentiality had extended outside of the hospital setting into a longer-term deferral of sexuality. Again, it might be argued that the reticence in addressing sexuality within a therapeutic setting has delayed rather than supported Owen's capacity to engage with his own vitality and recovery. This can be extended to a broader awareness of the kinds of background sensibilities through which sexuality is experienced. In the following extract, Joseph articulates a sense of what he has “lost” during his time in secure care, and the effects this has had upon his ability to engage in personal relationships:

Because it's been so long being out of a relationship. You don't, you can't get that-, what's it, you know pheromones between people, when you look at them and they're sort of pushing their aura on to you, as if to say, “This is me,” you know? Crazy that. But I didn't notice it, but [my support worker] did, and maybe it’s because I've been away from the, you know, dating that I don't pick up on the things, as they say, the little bits don't make sense.

What Joseph described is precisely the kind of “prereflective undirected bodily self-awareness” that Fuchs (2013, p. 2) sees as constituting vitality. He is no longer able to “pick up on” or read the signs that potential partners may be “pushing” onto him. This loss of a crucial aspect of his vitality went unnoticed, making it difficult to navigate the interactional dynamics of sexuality. For other patients this stripping away of parts of vitality was apparent during their time within inpatient care:

Well it makes you sort of immune to human contact in a way… If you've been in there for years and years, you can't go hugging the staff, do you know what I mean? There'll be days where you just want someone to give you a hug, and you can't go up to the staff and say, “Actually, these pills that you're going to give me are not going to do anything. What I really need is a hug.” And if you're going on for like for five, six, seven years like that, with no human contact, well it's just horrendous ‘cause then you just, it changes everything. Even now I would say, I still even in society, live by them sort of rules of, I'm very, I'm not a very touchy-feely person anymore because I've had to sort of train myself not to be. I suppose I could be alright now because I'm out and I'm not planning on going anywhere, but I'm forty-four now and I was like, what twenty-two, twenty-three at the time, so over all that time I've just learnt not to be touchy-feely… It's alright, but there's always still that element of, I still get, I get freaked out me, if people give me a hug. I get a bit tense and a bit like, “What are you doing?” almost looking round to see who's watching.

Stella reflected on her journey across secure care settings as a diminution of her capacity to experience and provide “human contact”. She described her gradual realization that the lack of touch from other people would ultimately “change everything” because she would have to “train” herself not to respond physically. But perhaps the most important reflection she made is that twenty years on from her first detention, she “could be alright now” because she does not expect her life to further change in any significant way (“I'm not going anywhere”). In effect, her growth as a person stalled in a significant way in her early twenties, and at 44, had stopped entirely. Stella shifted from living in a liminal hotspot to a situation we might describe, following Stenner (2017), as “permanent liminality”. She resigned herself to the paradox of potential. This need not have been the case. Matthew, for example, was able to gradually deparadoxify the lack of intimacy through physical contact with visitors, and subsequently with relationships formed during community visits:

Meeting someone just made me feel so complete and wanted, and … everything I was missing in my life, he made me feel like I had. The intimacy of it, just …. Not even sex side of it, just the intimacy, was just-, I never had. In hospital, you didn't even get a hug off anyone. If you were crying, you didn't get a hug. The only time you'd ever get a hug was every week when you had a visit… Just being cuddled up, entwined with someone a bit just made me feel so safe and secure and something that I hadn't felt ever.

Taking up opportunities provided by community visits is a well-understood aspect of secure care, typically acknowledged by ward staff. But patients also find opportunities to explore emergent practices within the hospital setting, through subversive means, such as intimate conversations held in plain sight but unheard by staff members:

I get to see that girl… I can to talk her, we go outside, we walk around and whatever… I've had mad conversations with her. She's sexually needy as well. It's like, if we could then we would, but we can't… You can't even be intimate. It's all got to be hush-hush. You couldn't talk the way we talk to each other, you couldn't talk like that in front of the staff.

**Fig. 2:**
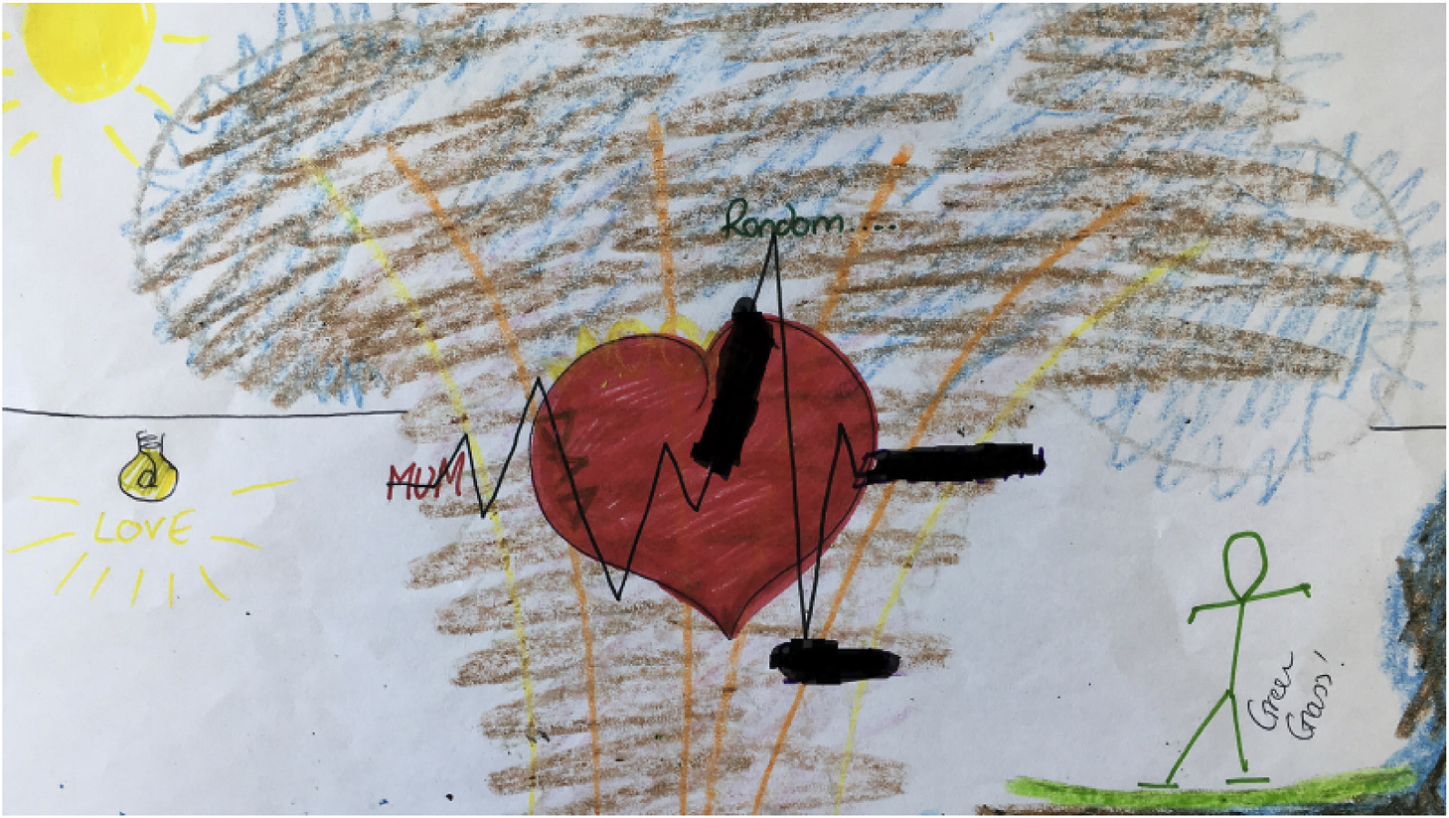
Claire's drawing].

The idea that “we would if we could” is pertinent here for Aaron. On one hand, it shows how sexuality can subvert, away from staff surveillance and into the hands of the patients, which provides an opportunity to access sexuality's life-giving potential (Fig. 2). Conversations fuel sexual imagery, which can then be activated in the privacy of the participant's bedroom. On the other, this activity demonstrates to the patients that they are still doing something illicit, potentially risky and, therefore, “wrong’“. The potential for any open and safe conversation about sexuality with staff is closed down and thus the possibility of intimacy is thwarted in favor of hushed conversations, which one could argue is riskier for those with complex histories (Ravenhill et al., 2020).

Sexuality can also be explored within the comparatively few private spaces that the secure settings offer, such as bedrooms. Emilie, a patient who heard voices, described her own emergent practice of finding a way to be sexual in a low secure setting:

Like do really … exotic dancing, like skinning their legs out, pulling their legs apart like that, going at the back of the legs, in and out, and then like that, you think about that, yeah. It's how they dance.

I: How often do you get chance to dance here, when you're at [hospital]?

I dance every night in my room… Every night. I put like lingerie on, and stockings and that, and I just stand around and dance in there on my own… I just put my music on, put lingerie on, and just like dance around, like twerk and that. With the voices that I'm listening to, that's, that's the way it sounds, like, that's what they enjoy. They like it when I do that. But Trevian^2^ is, like, a guy who fancies me. He wants me to be his girl. So I kind of hear him as a voice, he's now standing in my room by the bathroom door, and he's never going to go away. And he's there night and day, and I'm just like thinking, “Oh my God,” but he helps to get rid of the negative voices.

I: Right, I see.

So the voices that are bullying me, making me feel down, controlling me, things like that Trevian is kind of sticking up for me, being there for me, trying to make everything better. It makes me feel like … I'm active, I'm actually doing something, I'm not wasting time. Like, I'm not, there lying down, boring, not doing anything, it keeps me occupied, it keeps me motivated, and it just makes me feel a lot better to just like, do the little dance for a little while and then just go to bed… You're the first person who I told this. I don't know how they, what they would, if I should tell them that, that I dance around like a stripper in my room! ∗Laughs∗

Whereas Emilie is technically on her own in her room, her voice hearing experiences mean that, for her, she has an audience for whom she is performing. Through dancing provocatively for Trevian, Emilie feels able to manage her other voices, who are otherwise threatening, and experiences a sense of vitality and “feeling better” through being active and in movement. From a strictly clinical perspective, doubtless there might be concerns about whether Emilie's dancing is assisting her recovery, rather than serving to deparadoxify the liminal hotspot. But since staff appear to completely unaware of what Emilie does, there is no way for the institution to explore whether this practice enables some form of growth or not.

## 8 Discussion

A legitimate and highly pertinent response to the material we have presented would be to question why it is at all relevant to look at sexuality amongst mental health service users within secure and low-secure settings. Surely the need for detention within an inpatient setting indicates that a service user has experienced a level of crisis that is antithetical to forming or maintaining intimate relationships with others? And if it is taken for granted that sexual expressions are inevitable in these settings, why is the focus not exclusively on sexual safety and risk management? We hope that it is abundantly clear across the range of data we have analysed that service users experience sexuality as *both* a risk to their mental health and, *simultaneously*, an opportunity for growth. As such, sexuality is deeply connected with recovery, both during the time of inpatient and low-secure care, and in the longer-term hope for the possibility of forming relationships in the future. A recovery-oriented approach to mental health (which is the dominant model in the UK) then needs to find a way of addressing and engaging with sexuality and sexual desires.

We have described inpatient, and to a lesser degree low-secure care, as constituting a “liminal hotspot” (see Stenner et al., 2017), wherein there is an impasse between past crisis and future recovery. To navigate a liminal hotspot, the service user needs to find a way to “deparadoxify” the contradictory injunctions placed upon them, that they are both “unwell” and yet “recovering”. In this sense taking ownership of the idea that sexuality is both a risk *and* a powerful potential can be a way of managing the paradox and living through liminality because it is possible to see a transformed future. By contrast, linking current mental health to a suspension, or worse, a rejection of sexuality intensifies liminality and makes it more difficult to conceive of a future where relationships will again be possible. As some of the extracts show, this can result in a form of permanent liminality around sexuality, where service users find themselves locked in trying to resolve their paradoxical relationship to intimacy long after they have been discharged from inpatient care. This, ultimately, undermines recovery and leaves service users ill-prepared for resuming an independent life in the community.

A key issue here is in the ways that sexuality becomes objectified. Like any other institutional practice, secure hospital care inevitably objectifies aspects of experience which become targets for formal concern and intervention. We have shown that many service users have experienced “reductive objectifications”, where their sexual feelings or behaviors have been treated by clinicians and ward staff as directly indexed to their mental health and hence as either “too risky” or plain “wrong”. However, as Cussins (1996) argues, objectifications can be productive when they are placed within a broader life project. This would mean ensuring that all objectifications of sexuality are understood as fundamentally linked to recovery and only gaining a specific value and meaning when the specific, contextual implications for a particular service user's recovery journey are properly discussed and worked through. Such “a-signifying objectifications” may be as likely to result in the increasing of risk management as they are to the facilitation of sexual expression. The critical point here is that this cannot be known in advance of considering the specific issues and histories of the person(s) involved.

Many of the service users in the study described their own practices they had developed to manage the paradox of potential, from Claire's “surfing the urge”’ to Aaron's subversive “mad conversations” and Emilie's secretive dancing. It is particularly poignant that each of these service users was happy to share their experiences with us, as social researchers, but not with the ward staff or clinicians who might have been able to assist them in linking their experiences to their recovery. At a more significant level, this demonstrates that the management of sexual safety in these settings fails to engage with some of the very practices of which it is concerned. There is therefore greater rather than lesser risk involved when sexuality is subject to purely reductive objectifications which leaves service users both stuck with managing the paradox of potential on their own, and unwilling to discuss the things they are doing as a consequence. It also, as Ravenhill et al. (2020) point out, transfers risk to ward staff, as the persons who are most likely to have to decide on how to intervene when these practices become visible. It would be more appropriate for the institution to take ownership of risk around sexual safety through the development of comprehensive guidelines and procedures.

## 9 Conclusions – principles for the choreography of sexual safety

Based on the evidence we have presented in the current study and the related work of Ravenhill et al. (2020), Poole (2020) and Brown et al. (2014), we conclude with a series of principles which could ground guidelines and best practice discussions in secure mental health care. Primary amongst these is a call to recuperate sexuality from being understood within a narrowly defined notion of sexual safety, where the focus is entirely on the management of risk, and to restore the link to vitality and sexual wellbeing. It is manifestly clear that there are safeguarding issues around sexual expression and sexual behaviors in secure care, which institutions needs to develop clear policies and practices around (Brand et al., 2021; Quinn & Happell, 2016). But, as the material we have discussed demonstrates, a focus entirely on risk and safety is neither sufficient to engage with the emotions and actual sexual practices that service users engage in during their time within inpatient and low-secure care; nor does it enable consideration of the potential of sexuality for a recovery-oriented approach; and nor does it prepare people adequately for the reality of life after discharge (Bartlett et al., 2010). A more nuanced notion of “relational sexual safety” is required where safety is viewed as a shared practice in which service users and staff alike have a role.

Sexuality needs to be further included as an aspect of both initial assessments on entry to inpatient psychiatric care and explicitly addressed within care planning – a point made in earlier editions of the Royal College of Psychiatrists’s, 2017 report on sexual boundaries, but now removed. Despite the widespread use of formulation as an approach to assessment with the UK, it is rarely the case that service users are asked about their sexual histories. The message that sexuality is “unspeakable” and an obstacle rather than an enabler of recovery is then implicitly delivered very early during admission. Making sexuality a part of care planning, rather than a peripheral issue, would be a means of ensuring that all staff, from clinical and nursing staff to social work and health care assistants, would recognise responsibility in this area. This would help to address the situation described by Ravenhill et al. (2020) where ward-based staff defaulted to a view of sexual expressions of the part of service users as “organizational misbehavior” because they lacked appropriate policies and practice guidance. Again, this is likely to increase rather than decrease risk if service users felt that any discussion of sexuality is unwelcomed by staff.

As Tiwana et al. (2016) show in their review of policies around sexuality in psychiatric settings across Europe, there is not a binary choice to be made between a strict concern with sexual safety and a permissive embracing of sexual freedoms. Any policy will necessarily involve a deliberative practice where multiple stakeholders within the institution will be involved in conversations with service users about the boundaries and possibilities for behaviors (see Page et al. (2020) for an account of the use of a co-production model for discussing sexual safety in mental health wards). In one sense this is a further objectification of the lived experience of service users, where intimate aspects of their lives become a matter of institutional concern. But if those objectifications are choreographed as elements which by themselves have no a-priori signification but rather gain meaning when they are viewed within a consideration of the risks and potentials of sexuality for building key aspects of recovery such as relational intimacy, hope and vitality, they may lose much of their reductive nature. This further ties in with the importance we would place on the timing of any such discussion of sexuality. Just like the organic metaphor of the rose bud unopened presented by Josie, the timing of flowering is crucial, not to be imposed or prematurely forced, nor neglected for too long. Expanding this metaphor further, we would suggest instead that relational safety, developed through greater openness to issues of sexuality between staff and patients and thorough sexual and relationship history taking, might be serve to choreograph sexuality in a way that is more patient centred, timed exactly when the meanings that emerge with regards to sexuality can be unfolded safely and more specifically, relationally.

Liminality in its classic sense refers to a place or setting where a managed transition between two states of being occurs (van Gennep, 2019/1909). Entering psychiatric care – particularly as a young adult – can instead be experienced as admission to a liminal hotspot which then extends through time and space beyond the hospital and into community settings. The initial paradox of potential – “a rosebud, not open”, as Josie described it – may become a permanent experience of being caught between states whilst unable to properly realise growth and transition. Taking sexuality seriously in all its senses, with reference to safety, wellbeing, intimacy, relationships education and health, rather than a narrow focus on risk, offers the possibility of resolving or deparadoxifying both this liminal state and the wider barriers to growth and transformation within recovery.

## Declaration of competing interest

The authors declare that they have no known competing financial interests or personal relationships that could have appeared to influence the work reported in this paper.

## Footnotes

1 Occupants of such facilities are often referred to as “forensic” inpatients because they either have a history of offending and are under a criminal justice section or are believed to pose too great a risk of harm to themselves or others to be cared for in general psychiatric services. The people who took part in the present study are referred to as forensic inpatients.

2 Pseudonym for a rapper (musician) whose voice and presence Emilie reporting hearing and experiencing.
